# Variational Regression for Multi-Target Energy Disaggregation

**DOI:** 10.3390/s23042051

**Published:** 2023-02-11

**Authors:** Nikolaos Virtsionis Gkalinikis, Christoforos Nalmpantis, Dimitris Vrakas

**Affiliations:** School of Informatics, Aristotle University of Thessaloniki, 54124 Thesssaloniki, Greece

**Keywords:** non-intrusive load monitoring, energy disaggregation, NILM, deep learning, variational inference, multi-target regression, KL divergence, convolution neural networks

## Abstract

Non-intrusive load monitoring systems that are based on deep learning methods produce high-accuracy end use detection; however, they are mainly designed with the one vs. one strategy. This strategy dictates that one model is trained to disaggregate only one appliance, which is sub-optimal in production. Due to the high number of parameters and the different models, training and inference can be very costly. A promising solution to this problem is the design of an NILM system in which all the target appliances can be recognized by only one model. This paper suggests a novel multi-appliance power disaggregation model. The proposed architecture is a multi-target regression neural network consisting of two main parts. The first part is a variational encoder with convolutional layers, and the second part has multiple regression heads which share the encoder’s parameters. Considering the total consumption of an installation, the multi-regressor outputs the individual consumption of all the target appliances simultaneously. The experimental setup includes a comparative analysis against other multi- and single-target state-of-the-art models.

## 1. Introduction

Disaggregation is the process of breaking down a quantity into its separate elements. Specifically, the term energy disaggregation is a synonym for non-intrusive load monitoring (NILM) [1], a set of methods that aim to estimate the power consumption of electrical appliances that together compose the aggregate consumption of an installation. NILM can be thought as a blind source separation task [2], with only the mains consumption signal provided as input, and can be an essential tool for both individual consumers and distribution system operators (DSOs). From the consumer side, NILM constitutes a vital part of intelligent home systems, providing insights into reducing energy waste, raising energy awareness [3,4], improving the operational efficiency of installations [5,6,7], and creating smart alert mechanisms for residents in need [8,9,10]. On the other hand, DSOs can use NILM as a building block for various applications regarding the management and efficient monitoring of the grid [11,12] in combination with more accurate energy consumption forecasts [13,14]. In a similar fashion, disaggregation can be performed in other quantities that are used in residential buildings, such as natural gas [15] and potable water [16,17,18], in order to preserve resources and reduce the overall living costs of habitats.

Early attempts to confront the task of energy disaggregation used combinatorial methods to estimate the on/off events of each appliance [1] and Factorial Hidden Markov Models [19,20,21] to derive the appliance consumption. In FHMMS, a model consists of multiple independent HMMs and the output is essentially a combination of all the hidden states. During the last decade, deep learning solutions have come to dominate the energy disaggregation research field, producing state-of-the-art solutions [22]. Kelly and Knottenbelt [23] were the first to apply deep learning in order to tackle the problem of NILM, introducing three novel architectures. Subsequently, researchers in this field have devised a variety of solutions using different types of networks, including convolutional [24,25] and recurrent [26,27,28,29] networks as well as combinations of the two [30,31,32]. A number of these studies claim to have achieved state-of-the-art performance [24,27,31,33]. A popular technique from Natural Language Processing that produces good disaggregation models is the concept of attention [32,34,35,36]. The main idea is that the model can detect the most important parts of a sequence and learn to take them into consideration. Based on the results in the literature, this type of model shows great generalization capabilities. Moreover, the concept of data generation has been successfully applied to the problem of NILM as both a detection system [36,37,38,39] and a data generator [40,41].

Towards the direction of reproducible and comparable results, Symeonidis et al. [42] designed a framework composed of various stress test scenarios for evaluating energy disaggregation methods, whereas Batra et al. [43] implemented an easy-to-use API for rapid comparison of algorithmsalong with a set of baseline models. In an effort to standardize the way in which NILM experiments are conducted, Virtsionis Gkalinikis et al. [44] created Torch-NILM, the first Pytorch-based deep learning library for energy disaggregation. Torch-NILM contains tools to process time series data and build neural networks, along with three APIs to design experiments that follow an integrated benchmark method. Even though the aforementioned works are in the direction of standardization of experiments for tackling the comparability issue, at present the NILM research community lacks a globally accepted comparison framework [45].

The present article contributes to energy disaggregation research in the following ways. First, we present a novel neural network that is able to achieve multi-target disaggregation. The proposed network is built upon a combination of artificial layers such as CNN and fully connected layers [46], using the concept of variational inference in a similar way as in [47]. The proposed network is compared with a variation of the UNet-NILM multi-target model [31] and a baseline model. Additional experiments with known single-target models on the same data are included in order to measure the performance differences between the two strategies. Finally, we present the results of an ablation study to highlight the benefits of variational inference in the current task. The ablation analysis is essentially a comparison between the proposed neural network and a vanilla version without variational inference.

## 2. Related Work on Multi Target NILM

In an effort to provide more solid and deployable solutions towards practical NILM applications, multi-appliance approaches can be utilized. In this framework, one model accurately detects the electrical signatures of multiple targets. This results in the estimation of the corresponding individual energy usages simultaneously. Ideally, successful training should provide a model that automatically takes into account the energy allocation of all the targets and provides the right answer without any extra work. In this direction, Basu et al. [48,49] were the first to apply popular multi-label classification algorithms to the problem of NILM in order to detect on/off events with multiple targets. Furthermore, recent works have explored the concept of mixed-integer nonlinear programming (MINP) [50,51,52] to estimate the power consumption of many appliances simultaneously. These approaches are based on Hart’s original formulation [1] and perform disaggregation after modeling the power traces of the target appliances. Even though MINP solutions are lightweight and require few data for training, they are more suitable in cases where the data resolution is between 1 and 10 min.

In order to perform multi appliance disaggregation with deep learning, the general approach is usually composed of two steps: first, to detect the on/off events of the devices, and then to estimate their energy usage [53,54,55,56]. In a similar fashion, Verma et al. [57] faced the problem as a multi-classification task concerning on/off appliance states and addressed it by implementing an LSTM autoencoder. The proposed network was trained to compress the input into a latent feature space and reconstruct it with minimal information loss. Then, the latent features were used to perform multi-appliance disaggregation on the mains signal. Recently, Faustine et al. [31] followed a different direction and designed a multi-target model that outputs both the power time series and the on/off states of a set of target appliances simultaneously. The architecture is essentially a UNet model that consists of a series of 1D convolutional layers in combination with the idea of quantile regression. In the current paper, a novel architecture is designed with the capacity to output the power consumption time series of many appliances directly while keeping computational costs low. It should be noted that during our experiments we found that providing the on/off states of the appliances as ground truth to the model boosted its performance.

## 3. Practical Challenges in NILM

In terms of deep learning and neural networks, the majority of NILM research focuses on designing one network per appliance. While this strategy simplifies the problem and implementation, it has a number of drawbacks. To begin with, it is not a cost-effective solution for real-world applications deployed on cloud services, where the charge depends on the training duration and space requirements. A series of compression techniques suited for implementation on hardware with constrained computing capability were developed by Kukunuri et al. [58] in order to mitigate this problem and make the one vs. one strategy more usable in real-world solutions. Furthermore, a method of combining the results of all the models to provide a final answer needs to be designed, which is a non-trivial task. For instance, multiple models could detect the same end use (or part of it) as their target appliance. The choice of the correct output depends on a number of factors, such as the total consumption at the time of the specific end use, the general accuracy of the model, the uncertainty level of the answers, etc.

Apart from the deployment and practicality issues, the design of a NILM-oriented application should consider a number of hidden parameters. To begin with, a disaggregation algorithm heavily depends on the datasets used in its design and the evaluation. NILM algorithms aim to detect appliance events, which are closely affected by the habits and the culture of the users. Designers should take into account the fact that, although generalization capability of methods is a desirable property, the regionality of the data is an important factor that needs to be taken into account. Moreover, the sampling period of the data has a great effect on the detection limitations of algorithms. Usually, disaggregation research revolves around sampling periods of 1–10 s, which is a low sampling frequency in NILM. In lower granularities, e.g., 1–15 min, the unique features in electrical signatures vanish. As a result, accurate appliance event detection is impossible with such data. In these cases, nonlinear programming or combinatorial methods may be reasonable solutions to tackle the problem instead of more computationally intense deep learning models. On the other hand, neural networks can achieve state-of-the-art performance in situations where the granularity of the measurements is higher than 10 s. Finally, ensuring the reproducibility of NILM experiments is not an easy task, as there is no common bench-marking process among researchers. Thus, choosing a suitable NILM algorithm for an application is not always straightforward.

The current research concentrates on designing a deep learning architecture capable of detecting the desired set of electrical household appliances simultaneously. The network should be computationally efficient in order to be used in both commercial and research applications, and should have high training and inference speeds. The proposed architecture, which we call Variational Multi-Target Regressor (V.M.Regressor), has low storage and computation requirements, outperforms other multi-target disaggregation models, and is competitive with known state-of-the-art models that use the one vs. one (or single-target) strategy. Through an ablation experiment, we show how the key ingredients of the network increase its disaggregation capability compared to simpler implementations, whereas a comparison of three variants of the model indicates the best one. In order to identify any changes in performance of the models, the final experiment was designed using a different number of target appliances.

## 4. Materials and Methods

### 4.1. Datasets

The training for all the experiments of this work was executed on data from the UKDALE [59] public dataset, which contains data from five residential buildings in the UK. For evaluation of the models, we utilized data from theUKDALE and REFIT [60] datasets. These datasets are very popular among NILM researchers due to the fact that they contain high quality measurements with limited missing values. For most of the experiments, five household devices were chosen: a dishwasher (DW), fridge (FZ), kettle (KT), microwave (MW), and washing machine (WM). There were two main reasons for this. First, as these are widely used residential appliances, accurate disaggregation of their consumption could be of interest to users and DSOs. Second, these appliances have different operating characteristics, resulting in quite different electrical signatures. Thus, multi-target models need to extract the most useful and complicate characteristics in order to separate the individual sources and achieve high performance.

### 4.2. Data Preprocessing

In order for the neural networks to extract complex features and patterns, a minimum level of preprocessing should be applied to the raw data. The preprocessing of the data in this study comprised the following steps:

- Mains and target time series were aligned in time;
- Empty or missing values were replaced with zeros;
- Time series were normalised using standardization, with the values transformed centered around the zero mean with the unit standard deviation:
  (1) $$Z=\frac{x-mean}{std}$$
  where *Z* is the standard score, *x* is the datapoint, and *mean* and *std* the average and the standard deviation of the time series, respectively;
- The data were transformed in order to follow the sliding window method [27];
- The on/off states of the target appliances were calculated in each window. An appliance was considered to be working when its power level at the time of interest was above a predefined threshold. The power thresholds in this manuscript are drawn from the work of Kelly and Knottenbelt [23].

### 4.3. Methodology

The experiments reported in this work are summarized in Table 1. All the experiments were designed and performed using the Torch-NILM framework created by Virtsionis Gkalinikis et al. [44]. The same data preprocessing and model hyperparameters were used across all the experiments. All the models used the same input window with a length of 200 datapoints with the sliding window approach. The batch size was set to 1024 and the sampling period to 6 s. Each experiment was executed ten times on different random seeds on an Nvidia TitanX GPU.

In order to stress-test the methods under examination, the benchmark framework proposed by Symeonidis et al. [42] was loosely followed. This framework consists of four categories, each of which is an experimental scenario that aims to highlight the strengths and weaknesses of NILM detection algorithms. Category 1 of the benchmark framework involves performing training and inferencing on data from the same installation, whereas in Category 2 the algorithms are trained and tested on different buildings from the same dataset. The third and fourth scenarios evaluate the learning capabilities of the models across many installations in combination with inferencing on the same and or different datasets.

In the current research, the first two scenarios were executed as described in the original paper, whereas the third and the fourth were considered as one category and applied with a variation. Specifically, Category 3 in the experiments corresponds to training on only one dataset (UKDALE) and inference on the same (UKDALE) or another (REFIT). Hence, the ability of the models to learn across many houses was not evaluated in this case.

Table 2 summarizes the installations used for the benchmark categories for all the experiments in this study. The training period for UKDALE 1 was 5 months, from 1 March 2013 to 1 August 2013, whereas one month of data was used for inference in all scenarios.

### 4.4. Evaluation Metrics

A good NILM algorithm should have two qualities. First, it should successfully detect the operation states of the appliances. This is a non-trivial task, because overlapping events between different appliances is a common phenomenon and makes the detection more difficult. Second, the algorithm should provide good power estimation of the detected end uses. This is of high value, and is of concern to users and DSOs. As a result, the performance of NILM solutions should be evaluated with metrics that measure these properties.

In NILM research to date, two known machine learning metrics have typically been used. The performance in operation states detection is measured with $F_{1}$ (2), the harmonic mean of Precision (3) and Recall (4). High Precision indicates a low rate of false positives (*FP*), whereas high Recall means that the number of false negative (*FN*) is low. The $F_{1}$ score is a combination of these two. In Equations (3) and (4), the number of true positives is denoted as *TP*.

The ability to produce correct power estimations is measured using the *MAE*, which is calculated in Watts, as provided by (5), where *T* is the length of the predicted sequence, $y_{t}^{\prime }$ is the estimated electrical power consumption, and $y_{t}$ is the true value of active power consumption at moment *t*.

(2) $$F_{1}=2\frac{Precision\times Recall}{Precision+Recall}$$

(3) $$Precision=\frac{TP}{TP+FP}$$

(4) $$Recall=\frac{TP}{TP+FN}$$

(5) $$MAE=\frac{1}{T}\sum \;|y_{t}^{\prime }-y_{t}|$$

## 5. Topology of Neural Networks

In order to verify that the proposed solution has good performance, comparison with strong known baseline models is necessary. In the current case study, two main cases of performance comparison involved baseline models, namely, the benchmark for the multi-target detection models and the comparison between multi-target and single-target models. For the first case, two multi-target models were considered as the baseline, while one was used to achieve highly accurate results. In the second scenario, three known single-target architectures were chosen based on their performance and popularity.

An overview of the properties of the models used in the experiments is depicted in Table 3. It can be noted that the single-target models have more parameters in comparison to the multi-target architectures. Due to the fact that in this case one model corresponds to one appliance, the scenario of using many heavy algorithms for accurate disaggregation in production mode is unscalable. On the other hand, regarding the size of the networks, the multi-target algorithms are lighter, with decent training and inference times for multiple appliance disaggregation simultaneously. It should be noted that the same input window with a size of 200 was used for all tested appliances and models.

### 5.1. Single-Target Baseline Models

For the comparison of multi-target versus single-target strategies, three known NILM architectures were chosen: a denoising autoencoder, a sequence-to-point disaggregator, and a neural Fourier energy disaggregator. These models are all different from each other, and they were designed using different elements; thus, the comparative study should not be biased against similar architectures.

**Figure 1:**
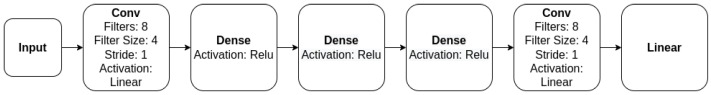
Architecture of DAE.

**Figure 2:**
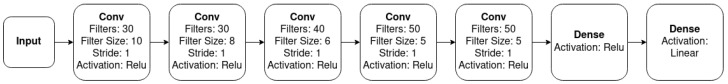
Architecture of S2P.

Denoising autoencoders are a family of neural networks designed to eliminate noise from input signals and output a clean signal. In NILM, the goal is to separate the appliance consumption from the mains consumption of the installation. Hence, the mains time series plays the role of the noisy signal, whereas the individual energy consumption is the noiseless target. The original architecture of DAE was proposed by Vincent et al. [61] and later adapted in NILM by Kelly and Knottenbelt [23] as a series of fully connected/dense artificial layers. The architecture is depicted in Figure 1:

The model called sequence-to-point (S2P) [24] is composed of a series of five convolutional layers that act as a feature extractor. These features are then passed through a dense layer with a ReLU activation layer. S2P is considered state-of-the-art, and is used across many papers in the literature either as an inspiration and/or as baseline. The architecture of the network is summarized in Figure 2.

**Figure 3:**
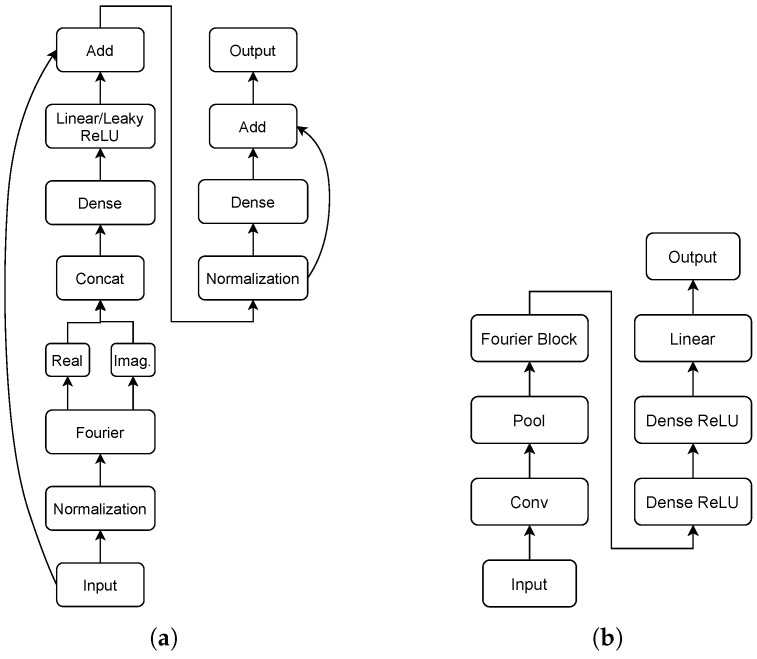
The NFED neural network: (**a**) Fourier block and (**b**) NFED architecture.

The Neural Fourier Energy Disaggregator (NFED) [62] could be considered as a member of the transformer family [63,64,65,66] due the fact that it is based on FNET [67], a transformer in which the attention layer is replaced by Fourier transformation as an efficient alternative. In comparison to state-of-the-art-models, NFED achieves similar performance with lower computational cost. NFED is composed of fully connected and normalised layers along with two main residual connections. The architecture is depicted in Figure 3.

### 5.2. Multi-Target Baseline Models

In order to evaluate the proposed implementation, an adaption of the UNet NILM proposed by Faustine et al. [31] was used. As input, the network receives the mains time series and outputs both the appliance states and power time series. In the original paper, UNet NILM performed quartile multi-target regression in a sequence-to-sequence fashion, with the length of the input being the same as the length of the output. Quartile regression involves smoothing of the mains and target time series using quartile filtering. This technique removes spikes and other features that may be valuable for successful disaggregation.

**Figure 4:**
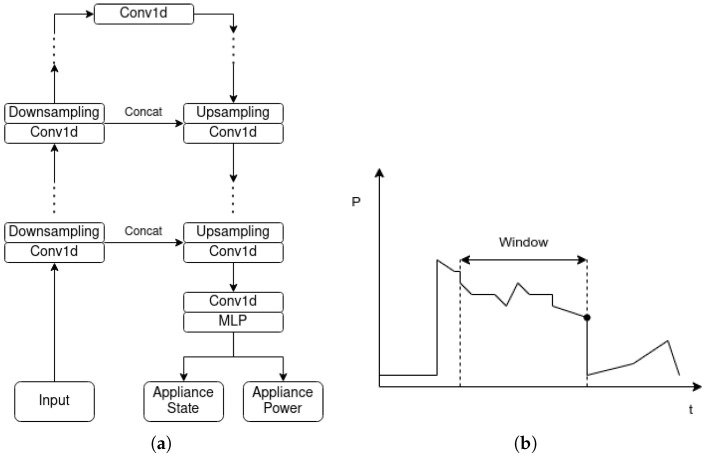
Architecture of UNet NILM: (**a**) UNet NILM and (**b**) sliding window approach.

Our variation of the UNet NILM differs from the original in the following aspects. First, we use the sliding window approach [27] instead of sequence-to-sequence. In this method, the input of the networks is a sequence and the output is the last disaggregated point of the sequence. Hence, near-real-time disaggregation can be achieved, along with faster training and inference. Second, regular regression without quartile smoothing was performed in order to compare the two implementations on the same level. Due to these changes, several parameters were adjusted in order for the model to perform at its best. The architecture of UNet NILM is depicted in Figure 4. Because the UNet NILM differs from the original implementation, the CNN-base architecture described in Faustine et al. [31] was used and adjusted accordingly in order to extract more insights about the performance of the models.

### 5.3. The Proposed Variational Multi-Target Regressor Architecture

**Figure 5:**
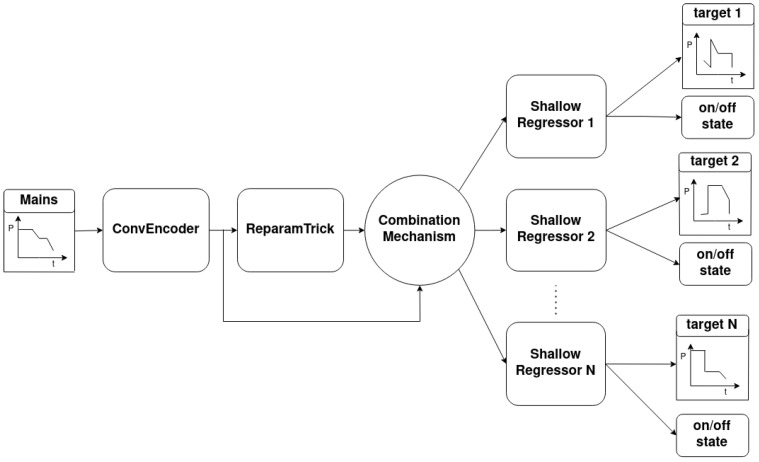
Architecture of Variational Multi-Target Regressor (V.M.Regressor).

**Figure 6:**
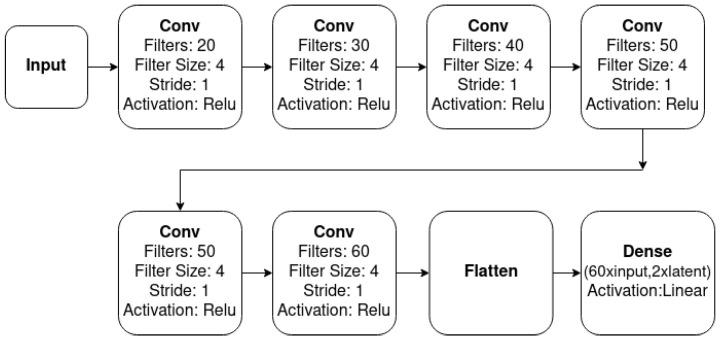
Architecture of ConvEncoder.

**Figure 7:**
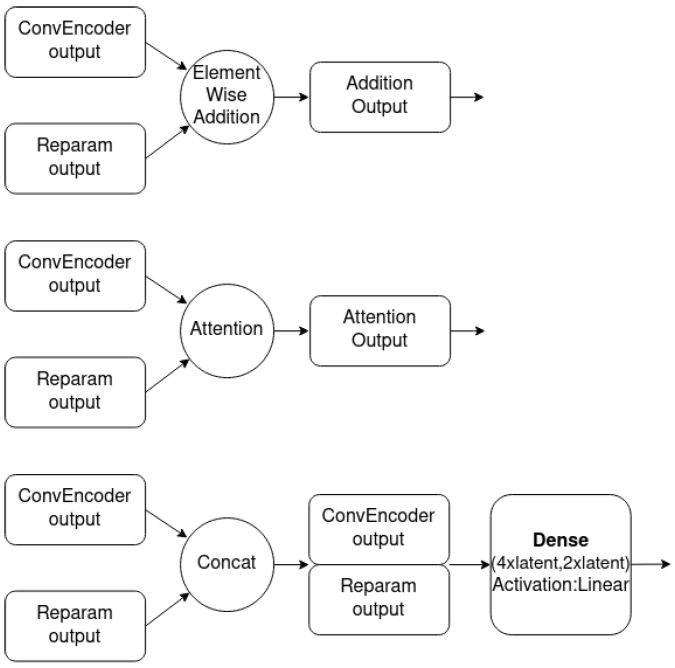
Overview of tested combination mechanisms.

**Figure 8:**
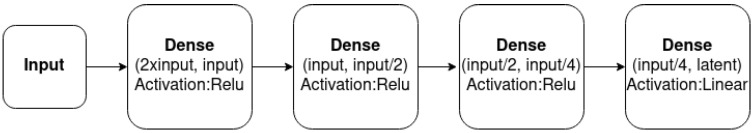
Architecture of Shallow Regressor.

**Figure 9:**
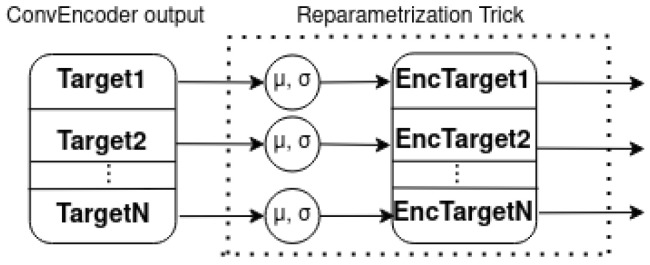
Reparametrization trick technique.

As depicted in Figure 5, the novel implemetation is a combination of four basic modules: the ConvEncoder model (Figure 6), the Combination Mechanism (Figure 7), the Shallow Regressor network (Figure 8) and the ReparamTrick module (Figure 9). After training, the model is supposed to output both the power consumption and the corresponding states of the target appliances.

The ConvEncoder is composed of a series of convolutional layers with the same kernel and different numbers of filters, which operate as a feature extractor. The output of the module passes through the ReparamTrick layer, where the sampling through the reparametrization trick is executed. Then, the two vectors are combined to produce a vector that contains the information from the extracted features and the encoding. The available combination mechanisms are depicted in Figure 7, and essentially produce a vector with a size equal to that of the ConvEncoder output. After observation during the designing of the architecture, combining the two vectors was found to boost the performance of the model.

In the current study, three lightweight and efficient combination methods were used. First, a simple element-wise addition of the two vectors was used. In this case, the addition acts as a residual connection [68] between the input of the ReparamTrick module. The idea is to provide the model with information extracted by the ConvEncoder in order to help in the training and fight any degradation issues [69]. Second, a dot attention mechanism [70] was used to help the model focus on the most significant parts of the two vectors. In addition, a dense neural layer with linear activation was trained in order for the model to learn to combine the vectors automatically.

Finally, the product of the combination mechanism is passed to all the ShallowRegressors to output the power and on/off estimation points for each target. Each ShallowRegressor is a series of lightweight fully connected layers that aim to filter out the unnecessary information and keep the valuable information regarding the corresponding target appliance.

The novel architecture is called Variational Multi-Target Regressor, because it uses the concept of variational inference [47,71] in order to boost the performance of a multi-target regression network. The intuition is that the network learns a posterior distribution instead of point estimates. The posterior distribution describes the target data more naturally than point estimates. Hence, the model is granted the ability to deal with unseen data points, resulting in more generalised predictions. Prior information is necessary to learn the posterior distribution, as described by Bayes’ rule (6), where given an input xϵR the unknown posterior p(z|x) is equal to the likelihood p(x|z) times the prior p(z) divided by the evidence p(x). This prior information is inserted as a hyperparameter, and aims to direct the model towards the right answer.

(6) $$p\left(z|x\right)=\frac{p(x|z)p(z)}{p(x)}$$

In NILM, these posterior distributions are based on many parameters, such as the electrical signatures of each appliance, the frequency of operation, the duration of end use, etc., and are generally hard to compute. As a result, an approximation of the posterior distribution should take place. The idea of variational inference dictates that the unknown posterior distribution can be approximated with another distribution q from the same family. Usually, the steps for this process are: (a) choosing a distribution family and (b) discovering the member of the family that is closest to the original data distribution. The distance between the posterior and approximate distributions is measured using the KL-divergence [72].

In order to successfully estimate the target distributions, the output of the encoder is divided by the number of targets into equal vectors, as shown in Figure 9. Then, the mean and standard deviation are learned for each target vector and, using the reparametrization trick, the corresponding encoded vectors are sampled. Then, with the same statistics, the KL-divergence for each target is computed. It should be noted that the proposed architecture as well as the various versions were implemented using Torch-NILM; the code is availabe at https://github.com/Virtsionis/torch-nilm, accessed on 17 January 2023.

### 5.4. Loss Function

As described earlier, the model approximates the posterior distribution, then estimates the power consumption and on/off appliance states of each target appliance. In order to insert all of this information into the training process, a new loss function was designed, presented in (7). This function consists of three different losses: the information loss, the regression loss, and the classification loss. Additionally, three normalization factors were used to scale each loss separately for best performance. For all the experiments, the values of beta, gamma, and delta are 0.001, 1, and 10, respectively.

The information loss (8) is the sum of the KL-divergence between the posterior approximation *q* and the prior *p* for each target divided by the number of appliances *N*, and is responsible for the posterior approximation. For the regression loss (9), the sum of all the mean square errors between the targets and the ground truths is used, scaled by the number of appliances. Similarly, the binary cross-entropy is calculated as the classification of the on/off states (10).

(7) Loss=beta∗info_loss+gamma∗class_loss+delta∗reg_loss

(8) $$info\_loss=\frac{1}{N}\sum_{n=1}^{N}KL(q_{i}\left(z|x\right)||p_{i}\left(z\right))$$

(9) $$reg\_loss=\frac{1}{N}\sum_{n=1}^{N}MSE\left(power_{i},power_{i}^{\prime }\right)$$

(10) $$class\_loss=\frac{1}{N}\sum_{n=1}^{N}binary\_cross\_entropy\left(state_{i},state_{i}^{\prime }\right)$$

## 6. Experimental Results and Discussion

This section contains five experiments. To begin with, an ablation study is executed in order to verify that the variational inference approach boosts the performance of a vanilla multi-target regression model. In addition, a performance comparison between three variations of the proposed network is conducted to determine the best one. Next, in an effort to highlight the capabilities of the proposed network, benchmarking comparisons with two multi-target and three single-target architectures are conducted. Finally, an investigation regarding the relation between the model performance and the number of target appliances is performed.

### 6.1. Ablation Study—Variational Inference

The goal of this investigation is to discover whether the variational inference approach assists the learning process of the proposed model. As a consequence, all the variations of the proposed model were compared side by side with the same network without the variational inference part. This model is called Vanilla, and skips the ReparamTrick and Combination Mechanism modules. Thus, the output of the ConvEncoder is directly passed to the ShallowRegressors. For this experiment, the first two categories of the benchmark were applied, with data from UKDALE used for training and inference.

**Figure 10:**
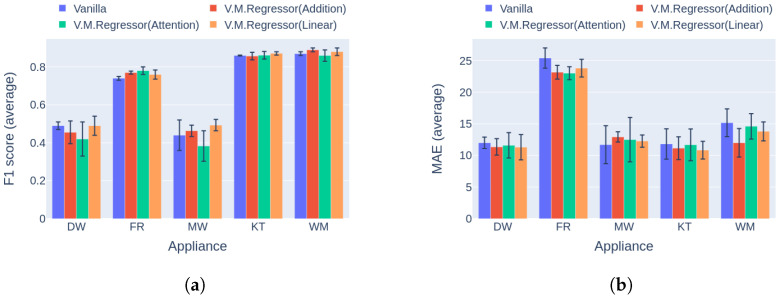
Experiment 1: Ablation Study, highlighting the effect of variational inference on the performance of the model. (**a**) F1 score in Category 1: single building NILM; (**b**) MAE in Category 1: single building NILM.

**Figure 11:**
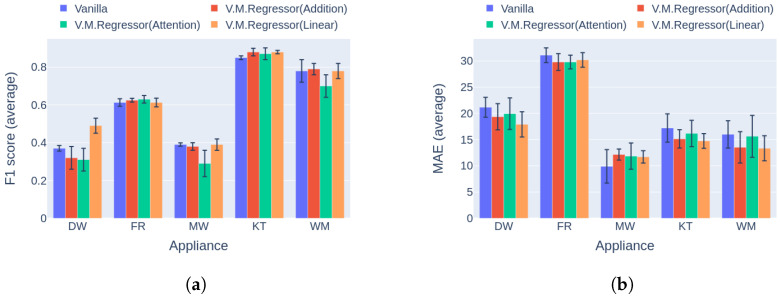
Experiment 1: Ablation Study, highlighting the effect of variational inference on the performance of the model. (**a**) F1 score in Category 2: training and inference on different buildings from UKDALE dataset; (**b**) MAE in Category 2: training and inference on different buildings from UKDALE dataset.

Regarding event detection, the results in Figure 10a and Figure 11a indicate that the proposed solution outperforms the vanilla variation in almost every case. In addition, the networks show better generalization capability on the unseen data following the variational inference (Figure 11a), reaching up to 27.9% higher performance compared to the vanilla implementation. The only cases in which the vanilla model achieves similar performance to the proposed counterpart are the dishwasher in Category 1 and the microwave in Category 2. Furthermore, the results in terms of the power estimation in Figure 10b and Figure 11b demonstrate that the proposed solution achieves lower MAE errors in 9 out of 10 cases, indicating better estimation ability than the vanilla model.

At this point it is useful to compare the models based on their computational cost. The properties of all the networks under investigation are depicted In Table 4. Comparing the Vanilla and the V.M.Regressor (Addition) models, it is obvious that the cost of integrating variational inference into the model is negligible. Additionally, the V.M.Regressor (Linear) model is slightly heavier than the vanilla model, with the addition of 100K parameters, which slightly affects the training speed of the model.

### 6.2. Combination Mechanism Selection

A crucial point in the novel architecture is the way in which the output of the ReparamTrick module is used. After experimentation, the novel architecture was implemented into three variations depending on the combination mechanism: simple addition mode, attention implementation, and a combination with a linear layer. In order to decide which mechanism was the best, the macro-level averages of the F1 score and MAE error were computed for the three categories of the benchmark. The macro-averaging process is essentially the simple averaging of the evaluation metrics across all the appliances. The results shown in Table 5 indicate that, for the first two categories, the model with the linear combination mechanism achieves the best performance. Regarding the scenarios in Category 3, the variant with the simple addition outperforms the other two. On the contrary, the architecture with attention shows the lowest performance in all scenarios. To highlight how close the overall performance of the variations with the linear and the addition modes are, the percentage differences between the averages of the two metrics per category are depicted in Table 6.

### 6.3. Comparison to Multi-Target Baseline

The third experiment was a direct comparison of the novel deep learning solution versus two multi-target architectures introduced by Faustine et al. [31], namely, the UNet NILM and CNN-Base networks. In the original implementation, UNet NILM achieved high performance in comparison to the CNN-Base model on experiments with the UKDALE dataset, and it is considered a strong opponent. In the current work, UNet NILM was adjusted to perform regular instead of quartile regression following the sliding window approach [27]. In this comparison, the best two variations of V.M.Regressor were used, that is, the versions with the addition and the linear combination mechanism.

**Figure 12:**
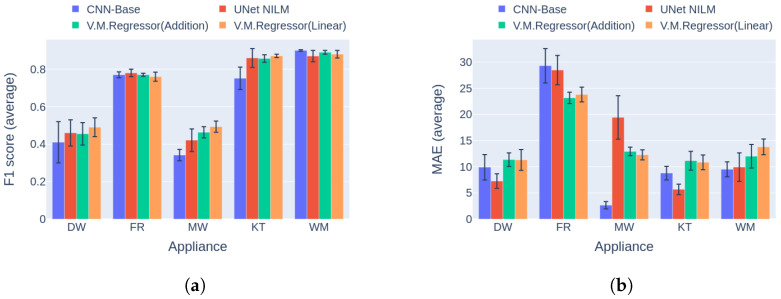
Experiment 3: Performance comparison of multi-target models in Category 1, with training and inference on UKDALE House 1. (**a**) F1 score in Category 1 (higher is better) and (**b**) MAE in Category 1 (lower is better).

In the first category of experiments, House 1 from the UKDALE dataset was used for training and inference. This category is the most usual case in real world applications, where a dedicated disaggregation model is trained for a residence. As can be seen in Figure 12, the V.M.Regressor (Linear) architecture reaches the maximum F1 score for three out of five target appliances, whereas for the remaining two there is a negligible difference between the state-of-the-art. On the other hand, in terms of the MAE error there is not a clear winner, with the UNet NILM ahead on three occasions and the rest of the models ahead on one appliance each. Even though V.M.Regressor is below the state-of-the-art in power estimation, it should be noted that the maximum absolute difference in MAE error is observed during disaggregation for the kettle, and is under 6 Watts. Considering the fact that a regular kettle operates at a maximum power level of around 2000 Watts, this difference is not very significant.

**Figure 13:**
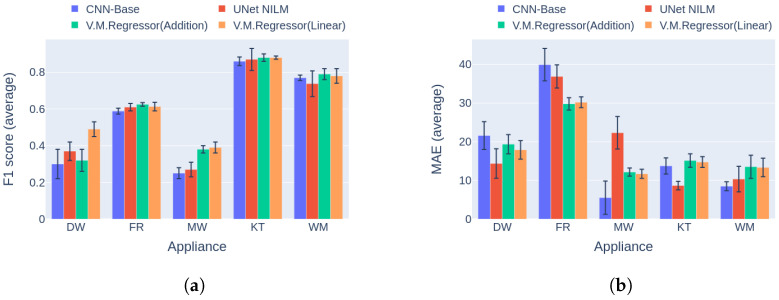
Experiment 3: Performance comparison of multi-target model in Category 2, with training on UKDALE House 1 and inference on UKDALE House 2. (**a**) F1 score in Category 2 (higher is better) and (**b**) MAE in Category 2 (lower is better).

The results in Category 2 are pictured in Figure 13, showing that V.M.Regressor (Linear) is the clear winner in terms of F1 score, with a 3.2% average difference across the five target appliances. On the appliance level, the largest differences in F1 score occur in dishwasher and microwave detection, with 6.3% and 9.5%, respectively. It should be noted that this category uses measurements from different installations of the same dataset for training and inference. Thus, an overall drop in performance is expected for many reasons, including the a great likelihood of different appliance models being in a house and the significant differences in the routines and habits of residents. This may explain the large drop performance of all models on washing machine and fridge disaggregation. A promising fact here is that the proposed model retains similar performance on the rest of the appliances. This highlights the fact that the generalization capability of the V.M.Regressor is better that the compared models. Regarding the power estimation and MAE error, there are mixed results. Specifically, the UNet NILM model achieves lower MAE errors for three appliances in comparison to V.M.Regressor, which performs better only on the fridge. In this case, the simple CNN-Base model performs better than the others on the microwave and the washing machine.

**Figure 14:**
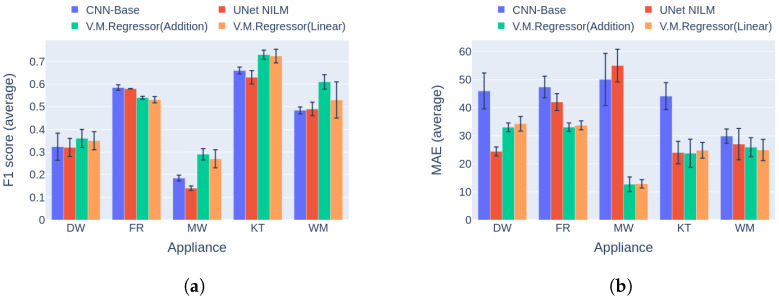
Experiment 3: Performance comparison of multi-target models in Category 3, with training on UKDALE House 1 and inference on REFIT House 2. (**a**) F1 score in Category 3 (higher is better) and (**b**) MAE on Category 3 (lower is better).

The experiments in Category 3 posed a greater challenge for the models. As in the previous category the training and inference were applied to data from different installations. The difference was that the installations were part of different datasets. This fact introduces many more possibilities and reasons for the models to underperform due to regionality, everyday habits, culture, etc. In this comparison, two use cases were explored. The first concerned training on UKDALE 1 and inference on REFIT 2 houses. The results of this scenario are depicted in Figure 14. It is notable that the V.M.Regressor variations are the clear winners on four out of five appliances with regard to both the F1 score and the MAE error. Yet again, the V.M.Regressor shows good generalization capacity, being able to out perform the competition.

**Figure 15:**
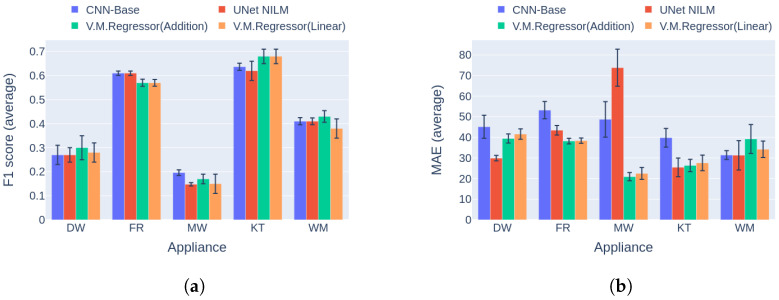
Experiment 3: Performance comparison of multi-target models in Category 3, with training on UKDALE House 1 and inference on REFIT House 5. (**a**) F1 score in Category 3 (higher is better) and (**b**) MAE in Category 3 (lower is better).

The second use case involves the same datasets while using the REFIT 5 house for inference. The results in this scenario are summarized in Figure 15. In this case, the novel neural network achieves the best event detection in three appliances, with the simple CNN-BASE winning in disaggregation of the fridge and the microwave. In the case of the MAE metric, V.M.Regressor had the lowest values on the fridge and the microwave, whereas UNet NILM performed better for the dishwasher and the washing machine. At the same time, UNet performed worse than the baseline model on microwave power estimation, producing the highest observed error across all of the experiments reported in this paper.

### 6.4. Comparison with Single-Target Models

Because the largest part of NILM research revolves around single-target solutions, it is useful to compare them with the proposed network. Specifically, the S2P architecture proposed by Zhang et al. [24] is considered to produce state-of-the-art performance, and has been used in almost every NILM paper as a strong baseline. The NFED [62] model is claimed to achieve similar performance using less computational resources. Finally, the DAE architecture [23] was one of the first architectures adjusted to solve the problem of energy disaggregation, and is included due to its popularity and high training and inference speeds. It should be noted that for this experiment the first two categories of the benchmark were used, with training and inference happening on data from the UKDALE dataset.

**Figure 16:**
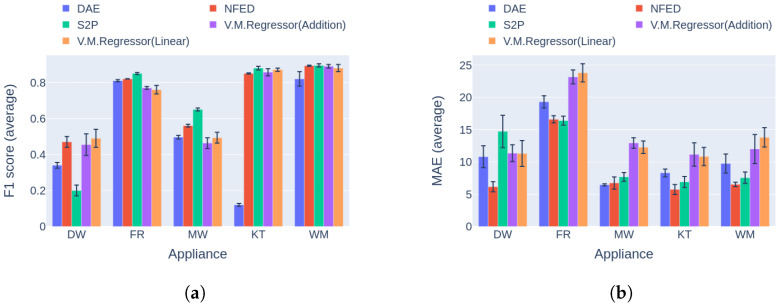
Experiment 4: Single-target vs. multi-target models in Category 1, with training and inference on UKDALE House 1: (**a**) F1 score in Category 1 (higher is better) and (**b**) MAE in Category 1 (lower is better).

The results for the first category are presented in Figure 16a. Regarding the F1 score, V.M.Regressor outperforms the single-target models in dishwasher disaggregation, whereas it achieves similar performance in event detection on the kettle and the washing machine. For the fridge and the microwavem the single-target models produce higher F1 score measures. In terms of the MAE error, the proposed model produces the highest values for four out of five of the appliances except the dishwasher. The clear winner in the MAE comparison is NFED, achieving the lowest errors for four out of five appliances. Although the model shows higher errors, the absolute differences are under 5 Watts except the case of the fridge. As a result, the proposed network could be easily applied to a practical NILM while providing results similar to single-target state-of-the art models, with a number of parameters almost 25 times lower and training time in case of five target appliances that is five times smaller, as shown in Table 3.

**Figure 17:**
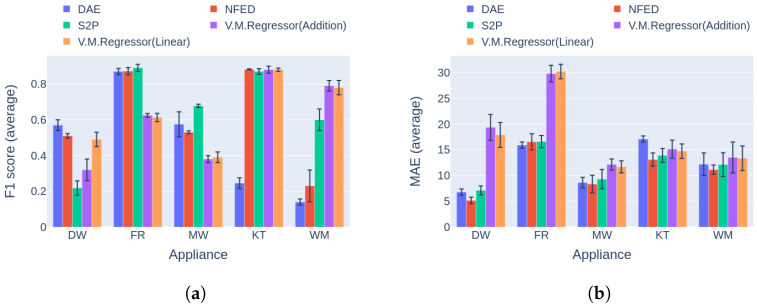
Experiment 4: Single-target vs. multi-target models in Category 2, with training on UKDALE 1 and inference on UKDALE 2 houses. (**a**) F1 score in Category 2 (higher is better) and (**b**) MAE in Category 2 (lower is better).

The final comparison in this experiment is based on the second category of benchmark [42]. After reviewing the results in Figure 17, it is obvious that V.M.Regressor outperforms the single-target models in terms of F1 score for the washing machine, with similar performance for the kettle and dishwasher. For the rest of the appliances, there is a large difference between the single-target models. It should be noted that none of the single-target models performs the same in disaggregating all the appliances. Hence, a different model could be more applicable for specific appliance disaggregation. On the aspect of power estimation, all models produce similar errors for three out of five appliances, except for the dishwasher and the fridge, where V.M.Regressor produces errors almost 15 Watts larger than the competition, reaching almost 30 Watts for the fridge. The newer types of fridges usually operate around 80–150 Watts, meaning that 30 Watts of deviation in power estimation is almost 20–38% of the total power level. On the other hand, a 20-Watt miscalculation of an average dishwasher end use corresponds to 5–10% of the operating power level, which is tolerable.

### 6.5. Performance for Different Numbers of Appliances

The number of appliances that the model can detect successfully is an important parameter of a practical NILM system. Thus, in the last experiments the performance of the proposed model and a baseline model were compared on different sets of appliances. For the set of two appliances, the kettle and the microwave were used. The set of four appliances contained the kettle, microwave, fridge, and washing machine. The set of six appliances included the dishwasher and the toaster. Finally, lights and an electric boiler were added to make up the set of eight devices.

**Figure 18:**
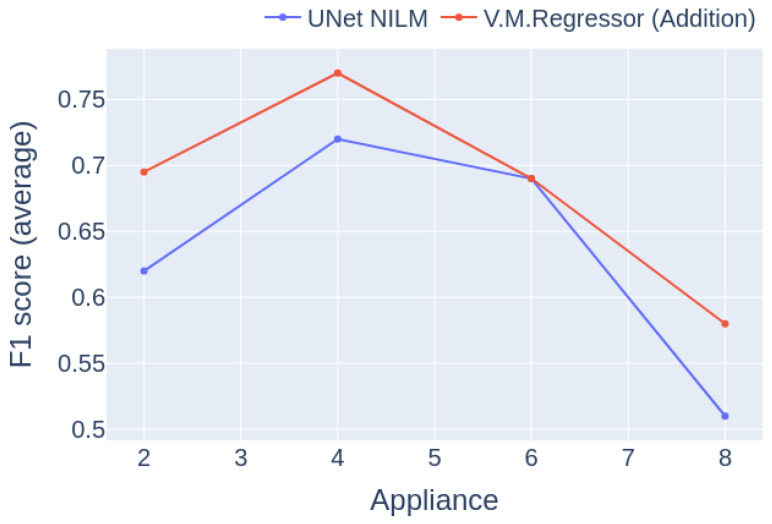
Experiment 5: Macro-F1 score per number of appliances for Category 1, with training and inference on UKDALE 1.

The experiment used data only from UKDALE house 1, with a training/inference ratio of 4/1. The results are presented in Figure 18. It is notable from the results that the performance of the models follows a similar trajectory; both reach the maximum at four appliances and the minimum at eight, whereas in case of simultaneous disaggreation of six appliances the curves intersect. It can be seen that the proposed model outperforms the baseline model in all cases.

## 7. Conclusions and Future Work

Applying deep learning networks to practical Non-Intrusive Load Monitoring applications is a non-trivial task. The root cause of the difficulty is that state-of-the-art architectures usually consist of a very large number of parameters. In addition, these networks are usually designed to disaggregate only one appliance at a time, meaning that the training and inference speeds and the overall size of the solution are heavily affected. Because these kinds of systems are usually built and operate on the cloud, a high costs are introduced.

In this article, we propose V.M.Regressor, a cutting edge deep learning architecture, as a solution for real world NILM systems. V.M.Regressor is capable of high quality simultaneous multi-target disaggregation with minimal computation requirements. Our proposed model outperforms a known state-of-the-art multi-target model of similar size, with faster training and inference speeds, and is competitive with heavier state-of-the-art single-target networks. The proposed model is build based on the principals of variational inference, which boosts its performance and the generalization capability on unseen data.

For future work, a number of suggestions can be made. To begin with, the concept of variational inference can be used to produce additional multi-target solutions. As the integration of this concept does not increase the number of the model parameters, it can be applied to boost the performance of lightweight architectures capable of running on embedded appliances. Training on many different datasets could be executed in order to increase the generalization ability of this type of model.

## Figures and Tables

**Table 1:** Summary of experiments.

Experiment	Environment Setup	Goal
Ablation study comparing the same network with and without variational inference.	Applied the first category of benchmarking [42], where training and inference happen during the same installation.	To highlight the performance boost due to the variational inference approach.
Performance comparison of three variations of the proposed network.	Applied the first two categories of benchmarking [42], where training and inference happen on installations from the same dataset.	To decide which combination method is the best.
Benchmark performance evaluation of multi-target models.	Executed the first three categories of benchmarking [42] for four installations from two different datasets.	For performance comparison of the novel model versus the baseline.
Performance comparison of multi-target models against single-target models.	Executed the first two categories of benchmarking [42], where training and inference happen on the same dataset.	For performance comparison of the novel multi-target model versus single-target baselines.
Performance comparison between multi-target models for different numbers of appliances.	Applied the first category of benchmarking [42], where training and inference happen on the same installation.	To highlight any performance boost or drop of the models.

**Table 2:** Installations used for the current study. UKDALE installation 1 was used for training in all categories, whereas UKDALE 1 and 2 were used for inference in Categories 1–2. REFIT installations 2 and 5 were used for evaluation in Category 3.

Appliance	Category 1		Category 2		Category 3	
	Train	Test	Train	Test	Train	Test
Dishwasher	1	1	1	2	1	2, 5
Microwave	1	1	1	2	1	2, 5
Fridge	1	1	1	2	1	2, 5
Kettle	1	1	1	2	1	2, 5
Washing Machine	1	1	1	2	1	2, 5

**Table 3:** Properties of the tested models: number of parameters, size of the model, training speed (GPU), inference speed (GPU and CPU). For the single-target models, the numbers are measured for experiments with one appliance. The input window was 200 datapoints for all appliances and models.

Strategy	Appliances	Architecture	Params (Mil)	Size (MB)	Training GPU (it/s)	Inference GPU (it/s)
Single-Target	1	DAE	2.9	11.540	102.13	139.20
		S2P	10.3	41.160	18.390	78.16
		NFED	4.7	18.956	20.220	44.93
Multi-Target	5	CNN-base	2.2	8.650	30.960	82.74
		UNet-NILM	2.2	8.940	14.750	50.01
		V.M.Regressor (Linear)	2.1	8.376	18.901	59.40
		V.M.Regressor (Addition)	2.0	8.170	19.405	61.10
		V.M.Regressor (Attention)	2.0	8.171	19.290	60.20

**Table 4:** Properties of the ablation study on combination methods for five appliances. Number of parameters in millions, size of the model on the disk, training speed (GPU), inference speed (GPU).

Architecture	Parameters (millions)	Size on the Disk (MB)	Training GPU (it/s)	Inference GPU (it/s)
Vanilla	2.0	8.170	19.8	62.2
V.M.Regressor (Addition)	2.0	8.170	19.4	61.1
V.M.Regressor (Attention)	2.0	8.171	19.3	60.2
V.M.Regressor (Linear)	2.1	8.376	18.9	59.4

**Table 5:** Experiment 2: Performance comparison between the available combination methods; the macro-average is the simple average of a metric across the five appliances. The best values are marked in bold. In terms of F1 Macro, higher is better. In terms of MAE Macro, lower is better.

Category	Train	Test	Combination	F1 Macro	MAE Macro
1	UKDALE 1	UKDALE 1	Addition	0.687	**14.118 **
			Attention	0.661	14.676
			Linear	**0.699**	14.402
2	UKDALE 1	UKDALE 2	Addition	0.599	17.993
			Attention	0.56	18.68
			Linear	**0.631**	**17.578**
3	UKDALE 1	REFIT 2	Addition	**0.506**	**25.678**
			Attention	0.446	29.402
			Linear	0.481	26.098
4	UKDALE 1	REFIT 5	Addition	**0.43**	**32.829**
			Attention	0.404	36.334
			Linear	0.412	**32.86**

**Table 6:** Percentage differences between the average macro-average scores for each category in Experiment 2. The best values are marked in bold. In terms of F1, higher is better. In terms of MAE, lower is better.

Category	Addition (F1)	Linear (F1)	F1 Diff (%)	Addition (MAE)	Linear (MAE)	MAE Diff (%)
1 & 2	0.643	**0.665 **	3.364	16.055	**15.99**	0.405
3	**0.493**	0.484	1.843	**29.254**	29.479	0.766

## Data Availability

Not applicable.
